# Matriptase Induction of Metalloproteinase‐Dependent Aggrecanolysis In Vitro and In Vivo: Promotion of Osteoarthritic Cartilage Damage by Multiple Mechanisms

**DOI:** 10.1002/art.40133

**Published:** 2017-07-05

**Authors:** David J. Wilkinson, Angela Habgood, Heather K. Lamb, Paul Thompson, Alastair R. Hawkins, Antoine Désilets, Richard Leduc, Torsten Steinmetzer, Maya Hammami, Melody S. Lee, Charles S. Craik, Sharon Watson, Hua Lin, Jennifer M. Milner, Andrew D. Rowan

**Affiliations:** ^1^ Newcastle University Newcastle upon Tyne UK; ^2^ Université de Sherbrooke Sherbrooke Quebec Canada; ^3^ Philipps University Marburg Marburg Germany; ^4^ University of California San Francisco

## Abstract

**Objective:**

To assess the ability of matriptase, a type II transmembrane serine proteinase, to promote aggrecan loss from the cartilage of patients with osteoarthritis (OA) and to determine whether its inhibition can prevent aggrecan loss and cartilage damage in experimental OA.

**Methods:**

Aggrecan release from human OA cartilage explants and human stem cell–derived cartilage discs was evaluated, and cartilage‐conditioned media were used for Western blotting. Gene expression was analyzed by real‐time polymerase chain reaction. Murine OA was induced by surgical destabilization of the medial meniscus, and matriptase inhibitors were administered via osmotic minipump or intraarticular injection. Cartilage damage was scored histologically and aggrecan cleavage was visualized immunohistochemically using specific neoepitope antibodies.

**Results:**

The addition of soluble recombinant matriptase promoted a time‐dependent release of aggrecan (and collagen) from OA cartilage, which was sensitive to metalloproteinase inhibition and protease‐activated receptor 2 antagonism. Although engineered human (normal) cartilage discs failed to release aggrecan following matriptase addition, both matrix metalloproteinase– and aggrecanase‐mediated cleavages of aggrecan were detected in human OA cartilage. Additionally, while matriptase did not directly degrade aggrecan, it promoted the accumulation of low‐density lipoprotein receptor–related protein 1 (LRP‐1) in conditioned media of the OA cartilage explants. Matriptase inhibition via neutralizing antibody or small molecule inhibitor significantly reduced cartilage damage scores in murine OA, which was associated with reduced generation of metalloproteinase‐mediated aggrecan cleavage.

**Conclusion:**

Matriptase potently induces the release of metalloproteinase‐generated aggrecan fragments as well as soluble LRP‐1 from OA cartilage. Therapeutic targeting of matriptase proteolytic activity reduces metalloproteinase activity, further suggesting that this serine proteinase may have potential as a disease‐modifying therapy in OA.

Osteoarthritis (OA) is the most common form of arthritis and a major cause of pain and disability in older adults. Although often considered a degenerative joint disease mediated by wear and tear and an inevitable consequence of aging, OA results from abnormal remodeling of joint tissues. Common risk factors include age, sex, prior joint injury, obesity, and mechanical factors. Moreover, there is now greater appreciation of the role of inflammation in human OA, and animal studies suggest that multiple, distinct biologic pathways contribute to disease initiation and progression in different OA models. Various mediators include double‐stranded RNA, Toll‐like receptors 1, 2, Wnt, transforming growth factor β, CXC chemokines, Indian hedgehog, alarmins, and intracellular zinc 3, 4. Such observations have led to the paradigm that during OA progression, chondrocytes undergo a phenotypic shift which detrimentally disturbs cartilage homeostasis.

Our findings in previous studies have implicated serine proteinases in the proteolytic cascades that lead to cartilage destruction 5, 6, 7, 8, and proteinase localized at the cell surface would be ideally placed to initiate the observed pericellular degradation of articular cartilage 9. In particular, we have reported a novel mechanism in OA whereby the type II transmembrane serine proteinase matriptase can drive cartilage collagenolysis by directly inducing and activating the pro forms of matrix metalloproteinases (MMPs) as well as by activating the G protein–coupled receptor, protease‐activated receptor 2 (PAR‐2) 10. PAR‐2, which can also be activated by several other serine proteinases, is expressed in numerous tissues 11 and has been demonstrated to be a pivotal player in mediating chronic joint inflammation 12. PAR‐2 deficiency is chondroprotective in the destabilization of the medial meniscus (DMM) murine model of OA 13, in which abnormal biomechanics trigger and potentiate OA 14. Recent findings confirm the high mechanosensitivity proteinase genes which are rapidly expressed following induction of experimental OA 15, and our own previous data confirmed elevated expression of both matriptase and PAR‐2 following DMM 10.

Taken together, these findings suggest that targeting PAR‐2 activators (which vary between tissues), rather than PAR‐2 directly, will help provide specificity and could therefore have potential as a disease‐modifying OA drug (DMOAD). Indeed, the proteolytic loss of type II collagen from cartilage is essentially irreversible 16, making such contributors to this process key therapeutic targets. Despite the well‐characterized involvement of metalloproteinases, including MMPs, inhibitors targeting this family of proteinases have not proven efficacious in arthritis 17.

The loss of the highly sulfated proteoglycan, aggrecan, from cartilage is considered to be an essential prerequisite for collagenolysis to ensue during disease 18, 19; ADAMTS enzymes, such as the mechanosensitive aggrecanase ADAMTS‐5 15, are widely implicated. Thus, blockade of aggrecanolysis could provide long‐term benefit to the integrity of the cartilage extracellular matrix.

The aim of the present study was to investigate the ability of matriptase to affect aggrecan release from human cartilage. We also sought to determine whether matriptase inhibition could reduce aggrecanolysis, and thus diminish the severity of cartilage damage, in murine OA.

## MATERIALS AND METHODS

### Reagents

All chemicals and reagents were of the highest purity available. Interleukin‐1α (IL‐1α) was a generous gift from Dr. Keith Ray (GlaxoSmithKline, Stevenage, UK). Oncostatin M (OSM) was produced in‐house as previously described 20, or purchased from R&D Systems. GM6001, a broad‐spectrum MMP inhibitor, was purchased from Calbiochem. ENMD‐1068 was from Enzo Life Sciences. ADAMTS‐5 was a kind gift from Professor Hideaki Nagase (Oxford, UK). MMP‐13 was produced as previously described 21. Bovine trypsin was from Sigma‐Aldrich. Purified full‐length human placental low‐density lipoprotein receptor–related protein 1 (LRP‐1) was from BioMac. Anti–LRP‐1 antibodies 8G1 (ab20384) and 5A6 (ab28320) were from Abcam, and neoepitope N‐terminal aggrecan fragment antibodies BC‐3 and BC‐14 were generously provided by Professor Bruce Caterson (Cardiff, UK). Antibodies to neoepitope C‐terminal aggrecan fragments (VDIPEN and NVTEGE), MMP‐13–cleaved collagen II neoepitope (GPHyGPQG), and the aggrecan G1 domain were kind gifts from Dr. John Mort (Montreal, Quebec, Canada). Anti‐matriptase antibody A11 was produced as previously described 22, and deglycosylated aggrecan was prepared as described 23. Boc‐Gln‐Ala‐Arg‐NHMec (Boc‐QAR‐NHMec) was from Bachem and Mca‐Lys‐Pro‐Leu‐Gly‐Leu‐Dpa‐Ala‐Arg‐NH_2_ (FS‐6) was from Sigma‐Aldrich. The matriptase inhibitor (S)‐3‐amino‐N‐(3‐(N‐(1‐(4‐(2‐aminoethyl)piperidin‐1‐yl)‐3‐(3‐carbamimidoylphenyl)‐1‐oxopropan‐2‐yl)sulfamoyl)phenyl)propanamide × 3HCl (referred to as compound 59 in ref. 24) was produced in‐house 24, 25 or bulk synthesized by Sygnature.

### Recombinant matriptase expression

Recombinant matriptase (catalytic domain; amino acids 596–855) was prepared at 4°C essentially as described previously 26. Following expression, pelleted bacterial cells (50 gm) were sonicated for 20 minutes (15 μm amplitude) in 2 × 450 ml of 0.1*M* Tris, 0.3*M* NaCl (pH 8.0) (buffer 1) while cooling in an ice/water mix, and then centrifuged at 10,000*g* for 40 minutes. Pellets were washed with buffer 1 before combining with 450 ml of 50 m*M* Tris, 6.0*M* urea, 1 m*M* β‐mercaptoethanol (pH 8.0) (buffer 2), sonicated, stirred for 15 minutes, and centrifuged at 10,000*g* for 60 minutes. The supernatant was applied to a ProBond column (50 ml; Life Technologies), equilibrated with buffer 2, and purified with His‐tagged protein, according to the instructions of the manufacturer. Matriptase‐containing fractions, identified by sodium dodecyl sulfate–polyacrylamide gel electrophoresis (SDS‐PAGE; Life Technologies), were pooled and dialyzed (each 5 liters) (once in 50 m*M* Tris, 3.0 *M* urea, 10% weight/volume [w/v] glycerol, 1 m*M* β‐mercaptoethanol [pH 9.0] and twice in 50 m*M* Tris, 1 m*M* β‐mercaptoethanol [pH 9.0] [buffer 3]). After dialysis, matriptase was purified on a MONO Q 10/100 GL column with an elution gradient of 0.0–1.0*M* NaCl in buffer 3. Fractions containing matriptase, identified by SDS‐PAGE (>95% purity), were pooled and stored at −80°C.

### Enzyme assays and inhibitor characterization

Active‐site titration of serine proteinases was performed using 4‐methylumbelliferyl 4‐guanidinobenzoate HCl. Serine proteinase activity assays were performed in 0.1*M* Tris HCl, 0.15*M* NaCl (pH 9.0), 500 μg/ml bovine serum albumin, 0.01% (w/v) Brij‐35, while MMP activity assays used 0.1*M* Tris, 0.15*M* NaCl, 10 m*M* CaCl_2_, 0.05% (w/v) Brij‐35, 0.1% (w/v) polyethylene glycol 6000 (pH 7.5). Serine proteinase and MMP activities were monitored by measurement of the increase in fluorescence from 50 μ*M* Boc‐QAR‐NHMec (excitation 360 nm; emission 460 nm) or 50 μ*M* FS‐6 (excitation 320 nm; emission 405 nm), respectively, at 37°C in an LS50B fluorometer (PerkinElmer); 4‐APMA was included in MMP assays to assess total MMP activity as previously described 27. The inhibitory property of compound 59 was verified by determination of the 50% inhibition concentration against matriptase, calculated from the percentage inhibition across a range of inhibitor concentrations. Dose‐response curves were generated using GraphPad Prism 5.0 software. *K*_i_ values were then determined using the Cheng‐Prusoff equation 28.

### Animals

Experiments were performed on 10‐week‐old male wild‐type C57BL/6J mice (weighing 25–30 gm). Mice were housed in standard cages with food and water available ad libitum (maintained in a thermoneutral environment). All procedures were performed in accordance with current UK Home Office regulations. OA in C57BL/6J mice was induced surgically by DMM; in a previous study, this resulted in medial and posterior rotation of the medial meniscus, leading to a mild form of OA 15.

Sham operations were performed in some animals. Antibody A11 was delivered via intraperitoneal osmotic pump (Alzet 1004) of 100 μl capacity, delivering 1.5 μg of antibody/day at a rate of 0.11 μl/hour for 4 weeks. Compound 59 was delivered at 5, 25, or 50 mg/kg/day via a subcutaneously implanted osmotic pump (Alzet 2004) of 200 μl capacity with a delivery rate of 0.25 μl/hour for 4 weeks. Both reagents were formulated in 0.9% saline, which was also used as vehicle‐only control. After 4 weeks, fresh osmotic pumps were inserted, with identical contents. Compound 59 was also administered to separate animals via 5 μl intraarticular injection of a 10 mg/ml solution (or saline alone as a control) at the time of surgery and again 1 week postsurgery. Mice treated with intraarticular injections or minipumps were killed 4 weeks or 8 weeks, respectively, after surgery, and knee joints harvested for histologic examination. Cartilage damage and osteophytes were graded under blinded conditions by 2 independent observers. according to previously described grading systems 29, 30.

### Immunohistochemistry

Decalcified murine knee joints were embedded in paraffin wax. Sections of joint tissue (6 μm) were deparaffinized and rehydrated, and antigen retrieval was performed using 1% (w/v) hyaluronidase (from bovine testes) incubated at 37°C for 30 minutes. Sections were then blocked with horse serum for 20 minutes and incubated with one of the following antibodies for 30 minutes at room temperature: anti‐VDIPEN (MMP‐cleaved aggrecan; 1:500 dilution), anti‐NVTEGE (ADAMTS‐cleaved aggrecan; 1:400 dilution), or anti‐GPHyGPQG (collagenase‐cleaved type‐II collagen; 1:400 dilution). After washing, sections were incubated with ImmPress Reagent (Vector) for 30 minutes. Signal was developed using 3,3′‐diaminobenzidine tetrahydrochloride (Dako) with hematoxylin counterstaining, according to the protocol recommended by the manufacturer (Dako). Images were captured using a 3‐CCD color video camera (JVC).

### Human cartilage

Macroscopically normal articular cartilage was obtained from patients with OA (age range 59–85 years; 92% female) who had undergone total knee replacement surgery in hospitals in Newcastle upon Tyne. The patients provided informed consent and the study was performed with ethics committee approval. Cartilage was dissected into ∼2 × 2 × 2–mm pieces, plated into 24‐well tissue culture plates (3 pieces/well, n = 4) in serum‐free medium, and incubated for 14 days in the presence of soluble recombinant matriptase (with or without inhibitors), with medium changed after 7 days, as previously described 10. For comparison purposes, some cartilage specimens were cultured with the potent catabolic stimulus of IL‐1 plus OSM, as previously reported 10.

Viability of cartilage explants was assessed by screening for adenylate kinase (AK) production using the ToxiLight Bioassay Kit (Lonza) 10. No increases in AK levels with any of the matriptase treatments were observed. Serum was excluded from explants since it can contain anabolic factors that can markedly alter the metabolism of cartilage; such exclusion does not affect tissue viability or responsiveness 31.

Cartilage discs derived from human mesenchymal stem cells (MSCs; Lonza) were generated as described 32 and used as a model of normal human cartilage. In all cartilage experiments, we used 100 n*M* matriptase, a concentration that was determined empirically to be optimal for the induction of human OA cartilage collagen and glycosaminoglycan (GAG) release (see Supplementary Figure 1, available on the *Arthritis & Rheumatology* web site at http://onlinelibrary.wiley.com/doi/10.1002/art.40133/abstract). Total RNA from cultured knee cartilage samples was prepared as described 7, or cartilage remaining at the final day of culture was digested with papain 31, and all samples were stored at −20°C until assayed. Measurements of hydroxyproline 33 and sulfated GAG 34 were used as estimates of cartilage collagen and aggrecan, respectively. Cumulative release was calculated and expressed as a percentage of the total for each well, as described previously 31.

### In vitro assays, SDS‐PAGE, and immunoblotting

Recombinant human matriptase (or bovine trypsin) was incubated with either aggrecan (1:40 enzyme:substrate ratio) or with purified LRP‐1 (1:5 enzyme:substrate ratio) in 0.1*M* Tris HCl (pH 8.5), 0.15*M* NaCl, 0.01% (w/v) Brij‐35 at 37°C. Products were separated by SDS‐PAGE and gels were stained with silver or semidry blotted onto PVDF. Aggrecan digests were probed with an antibody recognizing the G1 domain of aggrecan, while LRP‐1 immunoblotting was performed under nonreducing conditions in accordance with the instructions of the manufacturer. Reactivity was detected with enhanced chemiluminescence substrate and visualized using Genesnap photo capture software (Syngene). Since loading controls for cartilage conditioned media are not possible due to the absence of control proteins in serum‐free medium, equal volumes of conditioned media from human OA cartilage explants were resolved by SDS‐PAGE and subsequently immunoblotted.

### Real‐time polymerase chain reaction (PCR) of relative messenger RNA levels

For TaqMan PCR, relative gene expression was calculated using the ΔΔC_t_ method and corrected according to 18S ribosomal RNA levels. Cycling conditions (7900HT system; Applied Biosystems) for TaqMan PCR (TaqMan gene expression Master Mix; Applied Biosystems) were as follows: 10 minutes at 95°C, then 40 cycles of 15 seconds at 95°C, and 1 minute at 60°C. Human primer/probe sequences for TaqMan PCR have been previously described 35.

### Statistical analysis

Cartilage experiments were performed at least in quadruplicate for 3 different cartilage samples, and as >2 conditions were compared simultaneously, significance was assessed using analysis of variance with Bonferroni post hoc test for multiple comparisons, using GraphPad Prism 5.0 software. Standard TaqMan experiments were performed at least in triplicate on a minimum of 2 separate samples, with data analyzed using Student's 2‐tailed *t*‐test. Significant differences between cartilage damage scores of knee joints (typically n = 10 per treatment group) were assessed by Mann‐Whitney 2‐tailed U test, or, for experiments comparing >1 dose, a Kruskal‐Wallis nonparametric test with Dunn's multiple comparison test. For clarity, only selected comparisons are presented in some figures, where *P* values were <0.001, <0.01, or <0.05.

## RESULTS

### Aggrecan release from OA cartilage promoted by matriptase

**Figure 1:**
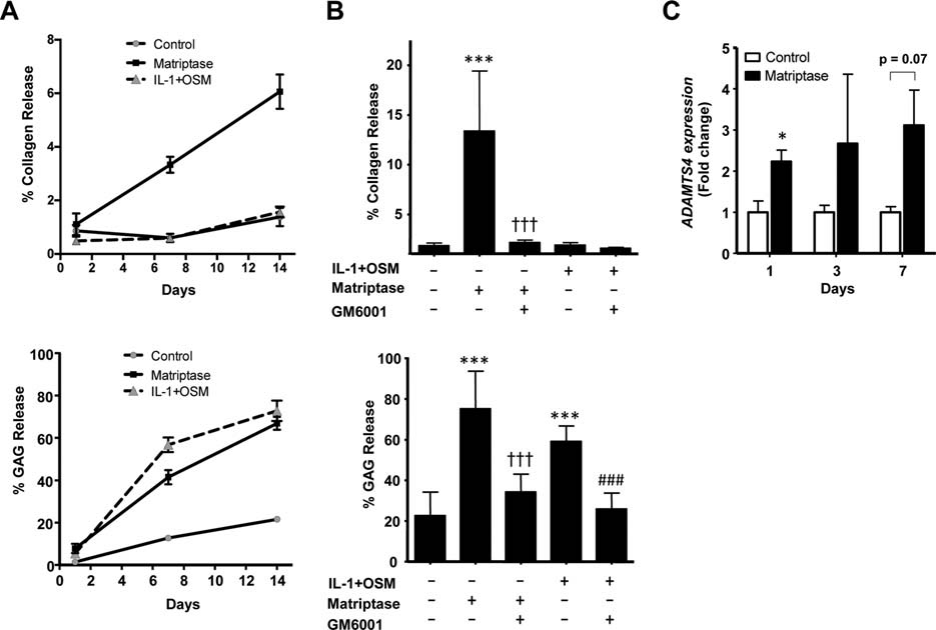
Promotion of aggrecan and collagen release from osteoarthritis (OA) cartilage by matriptase. Human OA cartilage explants were incubated with control medium or with interleukin‐1 (IL‐1) plus oncostatin M (OSM) (1 ng/ml and 10 ng/ml, respectively) or matriptase (100 n*M*) with or without GM6001 (10 μ*M*). Cultures were stimulated for 1, 7, or 14 days (**A**), 14 days (**B**), or 3 days (**C**). **A** and **B,** Collagen release (expressed as a percentage of the total collagen present) was determined by hydroxyproline measurement (top panels), and aggrecan release (expressed as a percentage of the total) was measured as glycosaminoglycan (GAG) release (bottom panels). Values are the mean ± SD (n = 4). ∗∗∗ = *P* < 0.001 versus control medium; ††† = *P* < 0.001 versus matriptase only; ### = *P* < 0.001 versus IL‐1 plus OSM only, by analysis of variance with Bonferroni post hoc test. **C**, For measurement of matriptase‐induced expression of ADAMTS proteinases, total RNA was isolated, reverse transcribed, and subjected to real‐time polymerase chain reaction. Results are relative expression levels normalized to the 18S ribosomal RNA housekeeping gene. Values are the mean ± SEM. ∗ = *P* < 0.05 versus control, by Student's 2‐tailed *t*‐test. All data in **A–C** are representative of 2–4 independent experiments.

Addition of recombinant matriptase to human OA cartilage in ex vivo culture over a 14‐day period resulted in a time‐dependent release of collagen, as previously reported 10, unlike the findings obtained with the potent pro‐catabolic stimulus IL‐1 plus OSM (Figure 1A). This effect was markedly blocked by inclusion of the metalloproteinase inhibitor GM6001 (Figure 1B). Release of aggrecan following matriptase stimulation was as pronounced as that observed with IL‐1 plus OSM stimulation (Figure 1A), and was again reduced to control levels in the presence of GM6001 (Figure 1B). Cartilage remained viable on day 14 in serum‐free medium as shown previously 5, and only IL‐1 plus OSM exhibited slight toxicity (<10%) (see Supplementary Figure 2, available on the *Arthritis & Rheumatology* web site at http://onlinelibrary.wiley.com/doi/10.1002/art.40133/abstract) 36. Furthermore, inclusion of the PAR‐2 antagonist ENMD‐1068 reduced aggrecan release (data not shown), as described previously 10. Finally, assessment of the expression of the ADAMTS aggrecanases *ADAMTS4* and *ADAMTS5* revealed a rapid, significant induction of *ADAMTS4* on day 1 following addition of matriptase to human OA cartilage. Although not significant, this trend continued to day 7 (Figure 1C); no alterations in *ADAMTS5* expression were observed.

**Figure 2:**
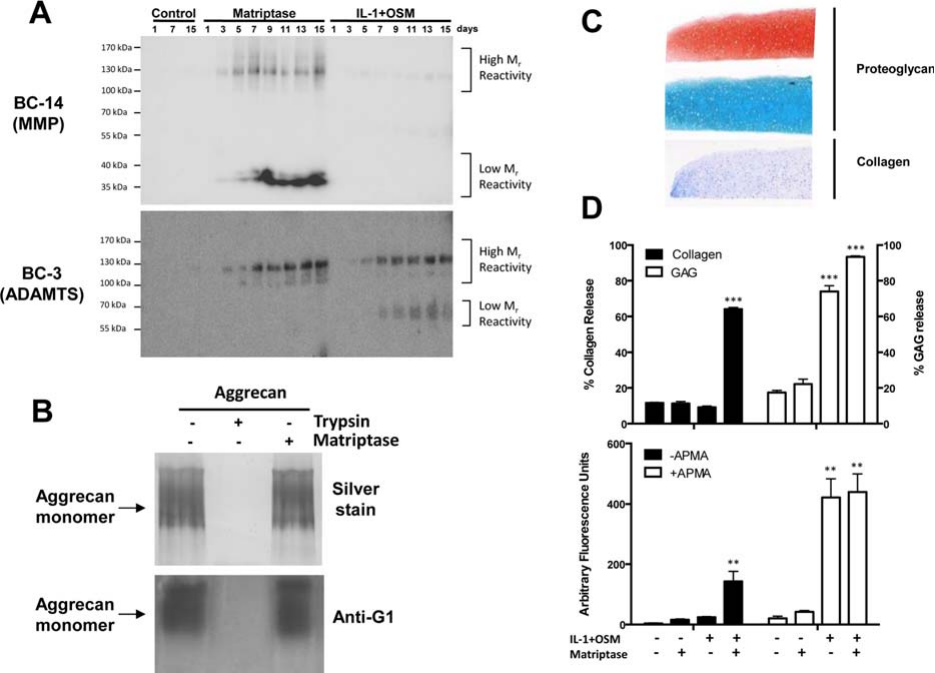
Promotion of matrix metalloproteinase (MMP)– and aggrecanase‐mediated aggrecan breakdown from OA cartilage by matriptase. Human OA cartilage explants were incubated with control medium with or without matriptase (100 n*M*) or IL‐1 plus OSM (1 ng/ml and 10 ng/ml, respectively). **A,** Western blotting of conditioned media (10% sodium dodecyl sulfate–polyacrylamide gel electrophoresis [SDS‐PAGE]) with the MMP‐specific neoepitope antibody BC‐14 (top) or the ADAMTS‐specific neoepitope antibody BC‐3 (bottom) was performed for the indicated numbers of days. The results demonstrate cumulative increases in both neoepiptopes over time with matriptase treatment, and in the ADAMTS‐specific epitope with IL‐1 plus OSM treatment. **B,** Deglycosylated aggrecan was incubated with matriptase or trypsin, and the resulting cleavage products were resolved by 7.5% SDS‐PAGE and visualized by silver staining (top) or Western blotting with an anti‐G1 antibody (bottom). **C**, Cartilage discs derived from human mesenchymal stem cells were assessed for proteoglycan content by staining with Safranin O (top) or Alcian blue (middle), and for collagen content by staining with Masson's trichrome (bottom). Original magnification × 5. **D,** After the discs were cultured in the presence of IL‐1 plus OSM and/or matriptase for 14 days, cumulative collagen release and aggrecan (GAG) release was measured (top). MMP activity (either active or total [pretreated with APMA]) in day 14 conditioned media was determined using the fluorogenic substrate FS‐6 (bottom). Values are the mean ± SD (n = 5 or more samples per group). ∗∗∗ = *P* < 0.001; ∗∗ = *P* < 0.01 versus control with no stimulation, by analysis of variance with Bonferroni post hoc test. All results are representative of 2–4 separate experiments. See Figure 1 for other definitions. Color figure can be viewed in the online issue, which is available at http://onlinelibrary.wiley.com/doi/10.1002/art.40133/abstract.

Time‐course data from studies of conditioned media from OA cartilage revealed that matriptase appeared to promote both MMP‐ and ADAMTS‐mediated cleavage of aggrecan within the interglobular domain, as evidenced by specific neoepitope antibodies (Figure 2A). Consistent with the lack of collagen release from human OA cartilage, IL‐1 plus OSM promoted only ADAMTS‐mediated cleavage and not that generated by active MMPs. Specificity of the neoepitope antibodies was confirmed using recombinant MMP‐13 and ADAMTS‐5 with deglycosylated aggrecan (data not shown). We also found that matriptase, unlike trypsin, failed to degrade the aggrecan monomer in vitro as evidenced by silver staining and anti‐G1 antibody staining (Figure 2B), suggesting little capacity to directly cleave the aggrecan core protein. To assess the ability of matriptase to promote aggrecan release from normal human cartilage, we used a stem cell–derived model (Figure 2C). We found that matriptase alone failed to significantly induce either collagen release (as previously reported) 10 or aggrecan release (Figure 2D). Conversely, IL‐1 plus OSM alone was able to promote significant aggrecan release, and in combination with matriptase also promoted the release of collagen, which was associated with the presence of active MMPs in the conditioned medium (Figure 2D).

### Release of LRP‐1 by matriptase

**Figure 3:**
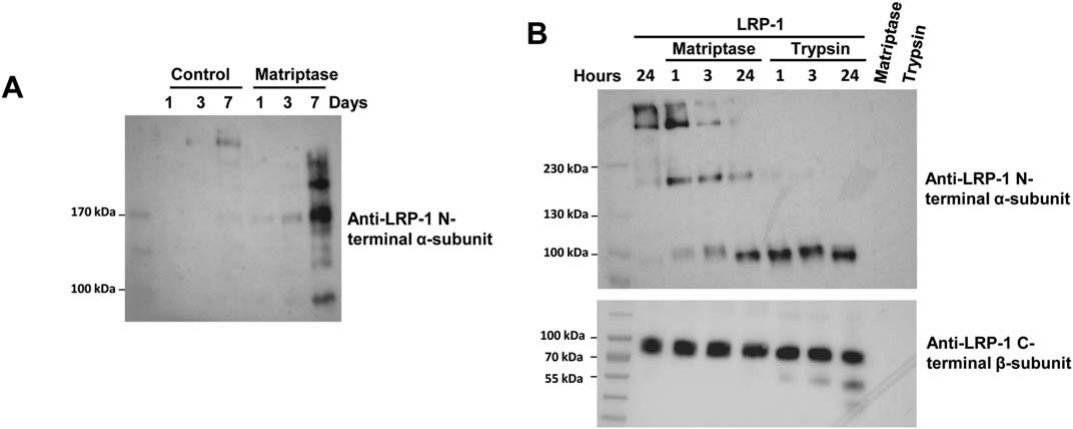
Matriptase cleavage of low‐density lipoprotein receptor–related protein 1 (LRP‐1). **A,** Conditioned media from unstimulated (control) and matriptase‐stimulated human osteoarthritis cartilage were harvested for the indicated number of days. Equal volumes of media (24 μl) were separated by 6% sodium dodecyl sulfate–polyacrylamide gel electrophoresis (SDS‐PAGE) and Western blotting with an anti–LRP‐1 antibody recognizing the N‐terminal domain. **B,** Matriptase was incubated with purified LRP‐1 in vitro at a 1:5 enzyme:substrate ratio for the indicated number of hours. Digested products were separated by either 6% or 4–15% SDS‐PAGE and Western blotting with anti–LRP‐1 antibodies recognizing the N‐terminal (top) and C‐terminal (bottom) domains, respectively. Results are representative of 3 independent experiments.

Since LRP‐1, an endocytic receptor for ADAMTS‐5, ADAMTS‐4, and MMP‐13 37, 38, 39, has been shown to be a key regulator of the proteolytic burden in cartilage, we hypothesized that matriptase could promote its release—a process likely to be important during cartilage destruction in OA 37. While low levels of high molecular weight LRP‐1 were detected in control cultures, as has also been observed by others previously (Yamamoto K: personal communication), addition of matriptase to human OA cartilage led to the detection of soluble fragments of the N‐terminal α subunit of LRP‐1 in day 7–conditioned medium (Figure 3A). This was not reduced following inclusion of the metalloproteinase inhibitor GM6001, and no LRP‐1 C‐terminal β subunit fragments were detected in these experiments (results not shown). To confirm cleavage by matriptase, purified LRP‐1 incubated with matriptase‐generated degradation fragments of the 515 kd α subunit of LRP‐1 (but not the 85 kd β subunit of LRP‐1) were detected, consistent with ex vivo findings (Figure 3B).

### Reduction of in vivo aggrecanolysis by inhibition of matriptase activity in experimental OA

**Figure 4:**
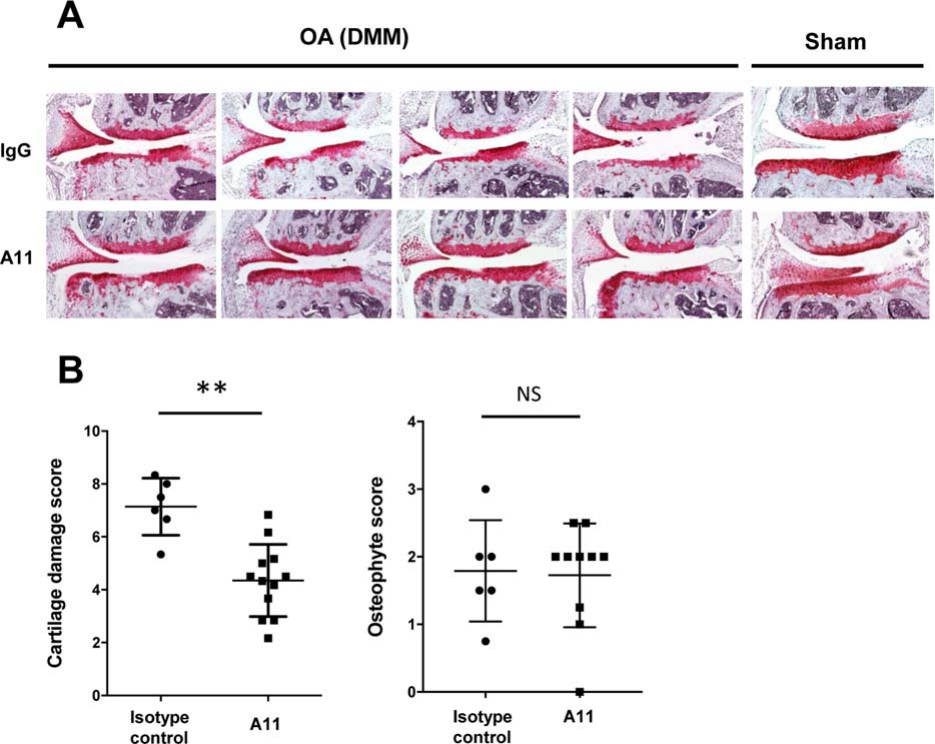
Matriptase‐neutralizing antibody protects against in vivo aggrecan loss in an osteoarthritis (OA) model. OA was induced in C57BL/6J mice following destabilization of the medial meniscus (DMM) surgery. Simultaneously, antibodies in 0.9% saline (either the A11 neutralizing matriptase antibody [n = 12] or an isotype control [n = 6]), at 1.5 μg/day were administered via intraperitoneal osmotic minipump. Pumps were replaced at 4 weeks, and animals were killed at 8 weeks postsurgery. **A**, Consecutive knee joint sections (6 μm) were stained with hematoxylin–Safranin O–fast green. Sections from 2 representative DMM‐treated animals and 1 representative sham‐treated animal in each antibody‐treated group are shown. Original magnification × 10. **B**, Approximately 10 sections from each mouse were graded by 2 independent observers who were blinded with regard to treatment group, using the following scoring systems: for cartilage damage, 0 (normal) to 6 (vertical clefts/erosion to the calcified cartilage extending to >75% of the articular surface) 29; for osteophyte size relative to the adjacent cartilage, 0 (none), 1 (small: similar thickness), 2 (medium: 1–3‐fold increased thickness), or 3 (large: >3‐fold increased thickness) 30. Bars show the mean ± SD of the highest scores in the medial tibial and femoral condyles for each joint. ∗∗ = *P* < 0.01, by Mann‐Whitney U test. NS = not significant. Color figure can be viewed in the online issue, which is available at http://onlinelibrary.wiley.com/doi/10.1002/art.40133/abstract.

**Figure 5:**
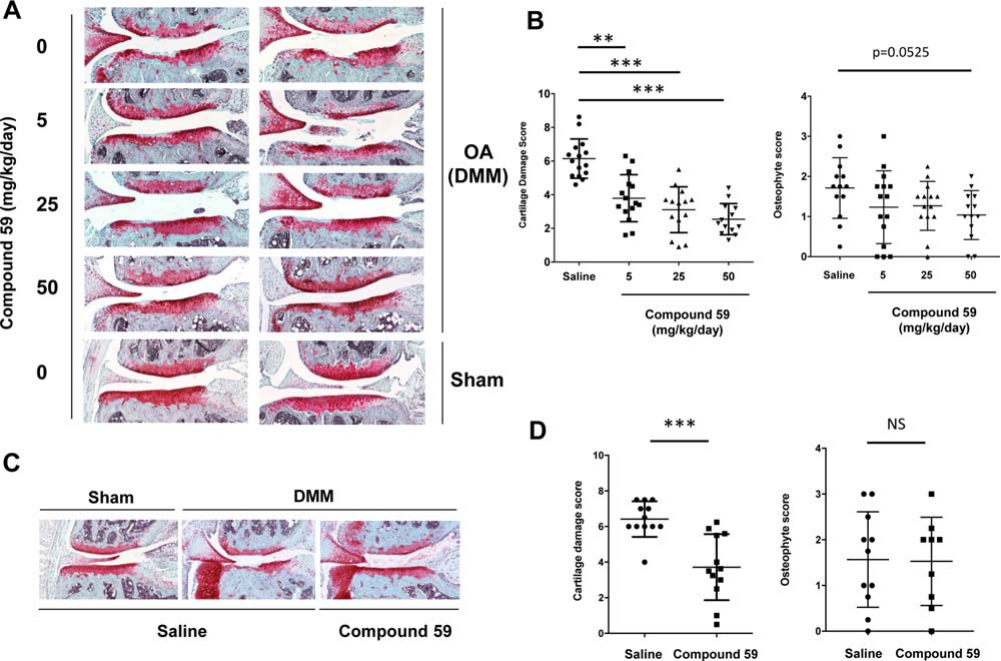
Matriptase inhibition protects against in vivo aggrecan loss in an osteoarthritis (OA) model. OA was induced in C57BL/6J mice following destabilization of the medial meniscus (DMM) surgery, while some animals underwent only sham surgery. Simultaneously, 0.9% saline alone (control; n = 15) or compound 59 in 0.9% saline at 5 mg/kg/day (n = 15), 25 mg/kg/day (n = 14), or 50 mg/kg/day (n = 13) was administered via subcutaneous osmotic minipump. Pumps were replaced at 4 weeks, and animals were killed at 8 weeks postsurgery (**A** and **B**). Alternatively, mice received 5‐μl intraarticular injections (10 mg/ml; n = 12 in both groups) on days 0 and 7 postsurgery (**C** and **D**). Consecutive knee joint sections (6 μm) were stained with hematoxylin–Safranin O–fast green. Images from a representative animal in each treatment group are shown. Original magnification × 10 (**A** and **C**). Sections from each mouse were graded (as described in Figure 4) by 2 independent observers who were blinded with regard to treatment group. Bars show the mean ± SD of the highest scores in the medial tibial and femoral condyles for each joint. ∗∗ = *P* < 0.01; ∗∗∗ = *P* < 0.001, by Kruskal‐Wallis test with Dunn's multiple comparison test (**B**) or by Mann‐Whitney U test (**D**). NS = not significant. Color figure can be viewed in the online issue, which is available at http://onlinelibrary.wiley.com/doi/10.1002/art.40133/abstract.

The neutralizing antibody A11 has been shown to be an effective matriptase inhibitor 22, and treatment of mice with A11 following OA induction by DMM reduced cartilage damage as evidenced by Safranin O staining (Figure 4A) and as shown by decreased cartilage damage scores (Figure 4B), while osteophyte grading showed no reduction in the size of osteophytes with A11 antibody treatment (Figure 4B). *K*_i_ determination data confirmed a very similar inhibitory profile for the small molecule matriptase inhibitor, compound 59 (*K*_i_ = 3.7 n*M*), as reported previously 24, while isothermal titration calorimetry measurements confirmed a 1:1 stoichiometry with high affinity (*K*_d_ = 20 n*M*) between compound 59 and matriptase (see Supplementary Figure 3 and Supplementary Table 1, available on the *Arthritis & Rheumatology* web site at http://onlinelibrary.wiley.com/doi/10.1002/art.40133/abstract). Compound 59 reduced aggrecan loss and cartilage damage scores even at the lowest dose used (Figures 5A and B). While there was a trend toward reduced osteophyte size with increasing dose of compound 59, this did not reach statistical significance (*P* = 0.0525 at 50 mg/kg/day) (Figure 5B). Furthermore, 2 intraarticular injections of compound 59 on days 0 and 7 post–DMM surgery were sufficient to confer significant protection against cartilage damage (Figures 5C and D), but no difference in osteophyte size was observed (Figure 5D). No cartilage damage was observed following intraarticular injection of saline in sham‐treated joints.

**Figure 6:**
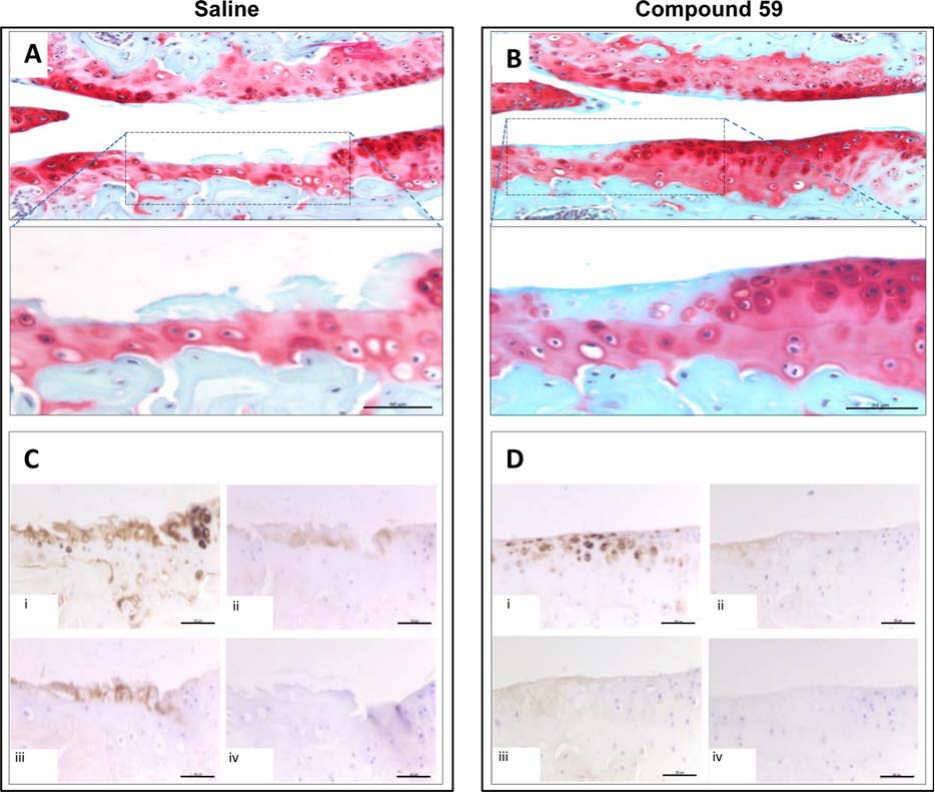
Matriptase inhibition blocks in vivo metalloproteinase activity in an osteoarthritis (OA) model. OA was induced in C57BL/6J mice following destabilization of the medial meniscus surgery. Simultaneously, 0.9% saline alone (**A** and **C**) or compound 59 in 0.9% saline at 50 mg/kg/day (**B** and **D**) was administered via subcutaneous osmotic minipump. Pumps were replaced at 4 weeks, and animals were killed at 8 weeks postsurgery. Consecutive knee joint sections (6 μm) were stained with hematoxylin–Safranin O–fast green. Sections were stained with neoepitope antibodies (**C** and **D**), showing matrix metalloproteinase–cleaved aggrecan sequence VDIPEN (i), ADAMTS‐cleaved aggrecan sequence NVTEGE (ii), or collagenase‐cleaved type II collagen sequence GPHyPGPQG (iii), or with no primary antibody (control) (iv). Images from a representative animal in each treatment group (n = 3 per group) are shown. Bars = 50 μm. In **A** and **B,** areas denoted by dashed lines in the upper panels are shown at higher magnification in the lower panels (original magnification × 20 and × 40, respectively).

We also observed no differences in osteophyte maturity between vehicle‐ and matriptase inhibitor–treated joints (results not shown). As previously reported 14, we found cartilage damage to the tibial plateau to be most marked following DMM (Figures 6A and B). Immunohistochemical assessment of DMM‐treated joint sections with 3 neoepitope antibodies recognizing ADAMTS‐cleaved (anti‐NVTEGE) and MMP‐cleaved (anti‐VDIPEN) aggrecan, as well as collagenase‐cleaved type II collagen (anti‐GPHyGPQG), confirmed that matriptase inhibition reduced antibody staining at or adjacent to sites of damage compared to that in saline‐treated control joints (Figures 6C and D).

## DISCUSSION

The discovery that distinct biologic pathways (for review, see refs. 3 and 4) are involved in OA initiation and progression represents major breakthroughs in the quest for effective therapies for this debilitating disease. Although there are currently no effective DMOADs, we are now beginning to better understand OA pathogenesis and, importantly, where such interventions could be effective. Our discovery of matriptase as a novel initiator of human cartilage degradation in OA represents one such pathway with therapeutic potential 10. In the present study, our findings demonstrated that matriptase is a potent inducer of aggrecan and collagen release from OA cartilage and that this is dependent upon metalloproteinase activity. The rapid release of aggrecan was associated with the generation of both MMP‐ and ADAMTS‐mediated cleavages of aggrecan unlike the potent cytokine stimulus of IL‐1 plus OSM which only affected ADAMTS‐mediated cleavages.

Moreover, multiple immunoreactive aggrecan fragments further highlight the potent consequences of matriptase action via numerous metalloproteinases. We hypothesize that although stimulation of human cartilage with IL‐1 plus OSM typically induces the expression and production of proMMPs 27, activation of such precursors to levels sufficient to drive collagenolysis does not occur in culture, as demonstrated by the absence of detectable collagen fragments from human OA‐ or MSC‐derived cartilage. Exogenous addition of the proMMP activator plasminogen is, however, sufficient to drive collagen release in, for example, IL‐1 plus OSM–stimulated human OA cartilage explants 5, and we have previously demonstrated the ability of matriptase to activate proMMPs and enhance cytokine‐stimulated cartilage breakdown 10, consistent with the data from the present study.

Interestingly, although matriptase is a trypsin‐like serine proteinase, it did not significantly degrade the aggrecan monomer in vitro, unlike trypsin itself. This suggests that the substrate specificity of matriptase is more restricted than that of trypsin, which is supported by previous studies 40, 41, such that the observed aggrecanolysis is essentially indirect by other proteinases. This was further supported by the finding that *ADAMTS4* (but not *ADAMTS5*) as well as MMPs 10 were induced following matriptase addition to OA cartilage.

The cell surface scavenger receptor LRP‐1 mediates cellular reuptake of several molecules that are important for maintaining cartilage homeostasis, including metalloproteinases such as ADAMTS‐5, MMP‐13, and ADAMTS‐4 37, 38, 39. It has been hypothesized that LRP‐1 “shedding” from the chondrocyte surface may play a key role in the accumulation of destructive proteinases during OA cartilage degradation in the absence of increased proteinase expression.

In the present study, we demonstrated for the first time that matriptase has the capacity to cleave LRP‐1 in both ex vivo and in vitro systems. The most common region for LRP‐1 ligand binding is cluster II, close to the N‐terminus (Yamamoto K: personal communication). Given that the 515‐kd fragment of LRP‐1 along with matriptase‐generated fragments of 200 kd and 100 kd were detected in vitro by Western blotting with an antibody specific for the N‐terminus, we conclude that if such cleavage occurs in vivo, cluster II would be released. Although these findings provide interesting insight into the potential “shedding” of LRP‐1 by matriptase, further studies are required to identify specific cleavage sites and confirm pathologic significance. Membrane‐bound MMP‐14 has been shown to shed LRP‐1 42, and our data clearly suggests that matriptase may also play a further indirect role in cartilage degradation via cleavage of LRP‐1, resulting in an accumulation of metalloproteinases and thus elevating the proteolytic burden within the extracellular matrix.

Our previous study demonstrated that PAR‐2 and matriptase are coexpressed in murine OA cartilage 10. The absence of cartilage damage in DMM‐treated PAR‐2–deficient mice further highlights the importance of PAR‐2 in murine OA 13, as well as in inflammatory arthritis 12, 43, 44. Moreover, we have previously reported that normal human cartilage fails to resorb following addition of exogenous matriptase, which was further supported in the present study by our use of MSC‐derived human cartilage. We hypothesize that this was due to PAR‐2 levels being below a critical threshold level, as indeed are (latent) MMP levels 10. Therapeutic targeting of PAR‐2 is being explored 43, although this may be hampered by the ubiquitous expression of this G‐protein–coupled receptor, which is thought to be critical in development, inflammation, immunity, and angiogenesis 44, as well as important in protecting musculoskeletal tissues from the destructive effects of inflammation and in promoting regeneration 45.

Since our ex vivo findings were made using only the recombinant catalytic domain of matriptase, and since inclusion of matriptase inhibitors abolished cartilage degradation (results not shown), we concluded that our observations are dependent on matriptase activity. Indeed, inhibition of matriptase activity as a potential therapeutic approach to treating OA was demonstrated using 2 different methods of inhibiting this transmembrane serine proteinase. We previously reported the potent inhibitory capacity of antibody A11 for matriptase 22, 46, and in the present study we demonstrated its ability to reduce cartilage damage when administered in vivo to DMM‐operated mice at a similar dosage as that used for a neutralizing PAR‐2 antibody study 13. Furthermore, both systemic administration and intraarticular delivery of a small molecule inhibitor of matriptase, compound 59 24, 25, significantly reduced cartilage damage in a dose‐dependent manner. Immunohistochemistry revealed this was likely due to reduced metalloproteinase activity (consistent with the use of GM6001 in matriptase‐stimulated human OA cartilage) as evidenced by reduced ADAMTS‐ and MMP‐mediated aggrecan cleavage as well as reduced collagenase‐mediated cleavage of type II collagen.

The relatively low level of staining for the aggrecanase‐mediated NVTEGE epitope is likely due to the concomitant MMP activity which effectively facilitates diffusion of the short ADAMTS/MMP‐generated aggrecan fragment (Phe^342^‐Glu^373^; human sequence numbering) 47 while leaving a residual G1 aggrecan monomer with the MMP‐generated VDIPEN C‐terminus. Although we did not confirm the presence of the Phe^342^‐Glu^373^ peptide from matriptase‐treated human OA cartilage, if it is present, this bioactive peptide could further contribute to cartilage breakdown via Toll‐like receptor 2 48. Together, these findings reveal that modulation of matriptase activity can regulate the in vivo proteolytic burden of OA cartilage.

We and others have previously demonstrated the ability of matriptase to activate several proMMPs 10 and have proposed that proMMP activation represents a key rate‐limiting step in cartilage destruction in disease 5, 6, 7, 21. The mechanisms by which matriptase is regulated in cartilage are unknown, although we have detected matriptase in murine cartilage following OA induction 10, which suggests that biomechanical mechanisms are important in this process. An intriguing feature of matriptase is that it automatically activates by a process that remains somewhat unclear 10, 49. This unusual property for a serine proteinase ideally places it as an initiator of proteolytic cascades, and our findings further support the concept that matriptase is a key proMMP activator in OA 10.

Osteophyte formation is a characteristic of OA, but a correlation with cartilage damage is not always apparent. For example, osteophytes in MMP‐13–deficient mice were no different in size at 8 weeks post‐DMM 30, and in our previous study, we found no osteophyte differences in DMM‐treated mice with exacerbated cartilage damage 29. Furthermore, results from our recent study showed reduced osteophyte size in PAR‐2–deficient mice following DMM, which are protected against cartilage damage 50, although an increase in osteophyte bone density was evident. With increasing doses of matriptase inhibitor we saw a trend toward reduced osteophyte size, suggesting perhaps that sufficient delivery of a potent matriptase inhibitor would also reduce osteophyte formation. Further research will be required to fully assess this latter possibility.

The cumulative impact of cartilage damage, which is perpetuated by multiple direct and indirect mechanisms over many years, is a significant contributing factor to OA progression and offers the potential for therapeutic intervention. Since aggrecanolysis appears to be a fundamental prerequisite for the essentially irreversible collagen breakdown 16, 19, there has been intense debate surrounding the most appropriate target for future therapy 51. In the present study, our findings demonstrate that matriptase indirectly promotes potent destruction of aggrecan via the action of both MMPs and aggrecanases, the levels of which may remain elevated in OA cartilage due to LRP‐1 release by matriptase. Finally, since effective inhibition of matriptase activity markedly reduces metalloproteinase activities and cartilage damage in vivo, we conclude that this serine proteinase is attractive therapeutically, and we suggest that targeting matriptase activity could serve as a potent, effective DMOAD.

## AUTHOR CONTRIBUTIONS

All authors were involved in drafting the article or revising it critically for important intellectual content, and all authors approved the final version to be published. Dr. Rowan had full access to all data in the study and takes responsibility for the integrity of the data and the accuracy of the data analysis.

### Study conception and design

Wilkinson, Leduc, Milner, Rowan.

### Acquisition of data

Wilkinson, Habgood, Lamb, Thompson, Hawkins, Désilets, Steinmetzer, Hammami, Lee, Craik, Watson, Lin, Milner, Rowan.

### Analysis and interpretation of data

Wilkinson, Hawkins, Lin, Milner, Rowan.

## Supporting information


**Supplementary Figure 1. Dose response for matriptase‐induced cartilage degradation.** Human OA cartilage explant cultures were prepared as described in Materials and Methods. Matriptase was added at different concentrations (1‐200 nM). Cartilage cultures were performed over 14 days, with re‐stimulation at day 7 with identical reagents. Remaining cartilage was digested with papain. Aggrecan (A) and collagen (B) breakdown were determined by GAG and hydroxyproline measurements, respectively, in both medium and digested cartilage. Data are presented as mean (± SD, n = 4) percentage release of the total for GAG and collagen, where statistical comparisons are ***, p<0.001 vs control (no matriptase); one‐way ANOVA with Bonferroni post‐hoc test.
**Supplementary Figure 2. Cartilage viability determination in 14‐day serum‐free explant culture.** Human OA cartilage was prepared as described in Materials and Methods. Explant cultures were stimulated with matriptase (100 nM) or IL‐1+OSM (1 and 10 ng/ml, respectively) for 1, 7 or 14 days. Medium from cartilage cultures was used in a ToxiLight Bioassay (according to the Manufacturer's instructions) to determine adenylate kinase levels within the conditioned medium. Some explants were killed by three freeze‐thaw cycles between −80°C and 37°C to represent 100% cell death; data are presented as mean (± SD, n = 4) percentage cell death compared to this positive control.
**Supplementary Figure 3. Isothermal titration calorimetry (ITC) analysis of compound 59 binding to matriptase at 25^**°**^C.** ITC was conducted essentially as previously described (Lamb HK, Mee C, Xu W, Liu L, Blond S, Cooper A, Charles IG, Hawkins AR. The affinity of a major Ca2+ binding site on GRP78 is differentially enhanced by ADP and ATP. J Biol Chem. 2006; 281:8796‐805). Briefly, buffer (50 mM Tris, pH 9.0, 1 mM β mercaptoethanol) containing 0.1 mM compound 59 was injected whilst stirring at 25^°^C into 11‐12 μM matriptase in the cell of a MicroCal VP‐ITC microcalorimeter (GE Healthcare). The first injection volume was 2 μl, followed by 24 injections of 10 μl. The experiment was repeated three times and data analysed using Origin Microcal software. **Upper panel**: Heat uptake upon injection (1 x 2 μl and 24 x 10 μl) of compound 59 (0.1 mM) into the calorimetric cell (1.4 ml) containing matriptase (12 μM). Heat pulses in the absence of compound 59 were negligible. **Lower panel**: Integrated heat pulses, normalised per mole of injectant, giving a differential binding curve that is adequately described by a single‐site binding model.
**Supplementary Table 1. Thermodynamic parameters for the binding of compound 59 to matriptase as measured by ITC at 25^**°**^C.** Shown are the values for *n*, the stoichiometry of binding; K_D(app)_, the apparent equilibrium dissociation constant; ΔH, the observed enthalpy; and ΔS entropy change for single site binding. The *c* values fall within the range of 1‐1000 that allows the isotherms to be accurately de‐convoluted with reasonable confidence to derive *K* values (Wiseman T, Williston S, Brandts JF, Lin LN. Rapid measurement of binding constants and heats of binding using a new titration calorimeter. Anal. Biochem. 1989;179:131‐137). Standard deviation (±SD) values are shown.
