# Supplementary material for: Matriptase Induction of Metalloproteinase‐Dependent Aggrecanolysis In Vitro and In Vivo: Promotion of Osteoarthritic Cartilage Damage by Multiple Mechanisms
Source: Arthritis Rheumatol. 2017 Jul 5;69(8):1601–11. doi: 10.1002/art.40133 (PMC5599990; doi:10.1002/art.40133)
Supplement: Supplementary file 1 — Supplementary Figure 1. Dose response for matriptase‐induced cartilage degradation. Human OA cartilage explant cultures were prepared as described in Materials and Methods. Matriptase was added at different concentrations (1‐200 nM). Cartilage cultures were performed over 14 days, with re‐stimulation at day 7 with identical reagents. Remaining cartilage was digested with papain. Aggrecan (A) and collagen (B) breakdown were determined by GAG and hydroxyproline measurements, respectively, in both medium and digested cartilage. Data are presented as mean (± SD, n = 4) percentage release of the total for GAG and collagen, where statistical comparisons are ***, p<0.001 vs control (no matriptase); one‐way ANOVA with Bonferroni post‐hoc test. Supplementary Figure 2. Cartilage viability determination in 14‐day serum‐free explant culture. Human OA cartilage was prepared as described in Materials and Methods. Explant cultures were stimulated with matriptase (100 nM) or IL‐1+OSM (1 and 10 ng/ml, respectively) for 1, 7 or 14 days. Medium from cartilage cultures was used in a ToxiLight Bioassay (according to the Manufacturer's instructions) to determine adenylate kinase levels within the conditioned medium. Some explants were killed by three freeze‐thaw cycles between −80°C and 37°C to represent 100% cell death; data are presented as mean (± SD, n = 4) percentage cell death compared to this positive control. Supplementary Figure 3. Isothermal titration calorimetry (ITC) analysis of compound 59 binding to matriptase at 25°C. ITC was conducted essentially as previously described (Lamb HK, Mee C, Xu W, Liu L, Blond S, Cooper A, Charles IG, Hawkins AR. The affinity of a major Ca2+ binding site on GRP78 is differentially enhanced by ADP and ATP. J Biol Chem. 2006; 281:8796‐805). Briefly, buffer (50 mM Tris, pH 9.0, 1 mM β mercaptoethanol) containing 0.1 mM compound 59 was injected whilst stirring at 25°C into 11‐12 μM matriptase in the cell of a MicroCal VP‐ITC microcalorimeter (GE H [file ART-69-1601-s001.docx]

**Supplementary Information**

**B**

**A**

**Supplementary Figure 1. Dose response for matriptase-induced cartilage degradation.** Human OA cartilage explant cultures were prepared as described in Materials and Methods. Matriptase was added at different concentrations (1-200 nM). Cartilage cultures were performed over 14 days, with re-stimulation at day 7 with identical reagents. Remaining cartilage was digested with papain. Aggrecan (A) and collagen (B) breakdown were determined by GAG and hydroxyproline measurements, respectively, in both medium and digested cartilage. Data are presented as mean (± SD, n = 4) percentage release of the total for GAG and collagen, where statistical comparisons are ***, p<0.001 vs control (no matriptase); one-way ANOVA with Bonferroni post-hoc test.

**Supplementary Figure 2. Cartilage viability determination in 14-day serum-free explant culture.**  Human OA cartilage was prepared as described in Materials and Methods. Explant cultures were stimulated with matriptase (100 nM) or IL-1+OSM (1 and 10 ng/ml, respectively) for 1, 7 or 14 days. Medium from cartilage cultures was used in a ToxiLight Bioassay (according to the Manufacturer’s instructions) to determine adenylate kinase levels within the conditioned medium. Some explants were killed by three freeze-thaw cycles between -80°C and 37°C to represent 100% cell death; data are presented as mean (± SD, n = 4) percentage cell death compared to this positive control.

**Supplementary Figure 3. Isothermal titration calorimetry (ITC) analysis of compound 59 binding to matriptase at 25^o^C.** ITC was conducted essentially as previously described (Lamb HK, Mee C, Xu W, Liu L, Blond S, Cooper A, Charles IG, Hawkins AR. The affinity of a major Ca2+ binding site on GRP78 is differentially enhanced by ADP and ATP. J Biol Chem. 2006; 281:8796-805). Briefly, buffer (50 mM Tris, pH 9.0, 1 mM β mercaptoethanol) containing 0.1 mM compound 59 was injected whilst stirring at 25^o^C into 11-12 μM matriptase in the cell of a MicroCal VP-ITC microcalorimeter (GE Healthcare). The first injection volume was 2 μl, followed by 24 injections of 10 μl. The experiment was repeated three times and data analysed using Origin Microcal software. **Upper panel:** Heat uptake upon injection (1 x 2 µl and 24 x 10 μl) of compound 59 (0.1 mM) into the calorimetric cell (1.4 ml) containing matriptase (12 µM). Heat pulses in the absence of compound 59 were negligible. **Lower panel:** Integrated heat pulses, normalised per mole of injectant, giving a differential binding curve that is adequately described by a single-site binding model.

**Supplementary Table 1. Thermodynamic parameters for the binding of compound 59 to matriptase as measured by ITC at 25^o^C.** Shown are the values for *n*, the stoichiometry of binding; K_D(app)_, the apparent equilibrium dissociation constant; ΔH, the observed enthalpy; and ΔS entropy change for single site binding. The *c* values fall within the range of 1-1000 that allows the isotherms to be accurately de-convoluted with reasonable confidence to derive *K* values (Wiseman T, Williston S, Brandts JF, Lin LN. Rapid measurement of binding constants and heats of binding using a new titration calorimeter. Anal. Biochem. 1989;179:131-137). Standard deviation (±SD) values are shown.
